# Analysis of the Impact of Cooling Lubricants on the Tensile Properties of FDM 3D Printed PLA and PLA+CF Materials

**DOI:** 10.3390/polym16152228

**Published:** 2024-08-05

**Authors:** Elvis Hozdić, Redžo Hasanagić

**Affiliations:** 1 Faculty of Mechanical Engineering, University of Novo Mesto, Na Loko 2, 8000 Novo Mesto, Slovenia; 2 Faculty of Technical Engineering, University of Bihać, Irfana Ljubijankića bb, 77000 Bihać, Bosnia and Herzegovina; redzo.hasanagic@unbi.ba

**Keywords:** additive manufacturing, emulsion, FDM, mechanical properties, PLA, PLA+CF

## Abstract

This study investigates the impact of infill density on the mechanical properties of fused deposition modeling (FDM) 3D-printed polylactic acid (PLA) and PLA reinforced with carbon fiber (PLA+CF) specimens, which hold industrial significance due to their applications in industries where mechanical robustness and durability are critical. Exposure to cooling lubricants is particularly relevant for environments where these materials are frequently subjected to cooling fluids, such as manufacturing plants and machine shops. This research aims to explore insights into the mechanical robustness and durability of these materials under realistic operating conditions, including prolonged exposure to cooling lubricants. Tensile tests were performed on PLA and PLA+CF specimens printed with varying infill densities (40%, 60%, 80%, and 100%). The specimens underwent tensile testing before and after exposure to cooling lubricants for 7 and 30 days, respectively. Mechanical properties such as tensile strength, maximum force, strain, and Young’s modulus were measured to evaluate the effects of infill density and lubricant exposure. Higher infill densities significantly increased tensile strength and maximum force for both PLA and PLA+CF specimens. PLA specimens showed an increase in tensile strength from 22.49 MPa at 40% infill density to 45.00 MPa at 100% infill density, representing a 100.09% enhancement. PLA+CF specimens exhibited an increase from 23.09 MPa to 42.54 MPa, marking an 84.27% improvement. After 30 days of lubricant exposure, the tensile strength of PLA specimens decreased by 15.56%, while PLA+CF specimens experienced an 18.60% reduction. Strain values exhibited minor fluctuations, indicating stable elasticity, and Young’s modulus improved significantly with higher infill densities, suggesting enhanced material stiffness. Increasing the infill density of FDM 3D-printed PLA and PLA+CF specimens significantly enhance their mechanical properties, even under prolonged exposure to cooling lubricants. These findings have significant implications for industrial applications, indicating that optimizing infill density can enhance the durability and performance of 3D-printed components. This study offers a robust foundation for further research and practical applications, highlighting the critical role of infill density in enhancing structural integrity and load-bearing capacity.

## 1. Introduction

Additive manufacturing (AM) is a transformative technology that fabricates products through a layer-by-layer addition of material, allowing for the production of complex geometries, rapid prototyping, and small batch flexibility [1,2,3,4,5,6]. This approach supports the creation of intricate geometries, rapid prototyping, and flexible small batch production, offering advantages such as reduced material waste, shortened production times, and the capability to use diverse materials within a single process, as exemplified in industries like aerospace and medical device manufacturing. However, AM faces challenges including anisotropic behavior and suboptimal mechanical properties of printed parts, which can limit their performance and application in load-bearing scenarios [7,8,9,10,11,12,13,14].

AM technologies can be categorized based on the type of materials used, which include polymers [15,16,17], metals [18,19,20,21], and ceramics [22,23,24,25,26], or based on the processing methods, such as material extrusion, powder bed fusion, and vat photopolymerization. Furthermore, AM technologies are distinguished by the initial form of the material, including liquid, filament, or powder [27]. Techniques like laminated object manufacturing (LOM), fused deposition modeling (FDM), selective laser sintering (SLS), stereolithography (SLA), direct energy deposition (DED), and inkjet printing (IJP) illustrate the diversity within AM methods. The overlap and variety of names for these technologies add complexity to their classification [27].

Among these technologies, fused deposition modeling (FDM) stands out as one of the most prevalent due to its simplicity, versatility, and broad adoption across various industries, including automotive, aerospace, and consumer products [14,28,29,30,31]. FDM works by extruding thermoplastic material through a heated nozzle, layer by layer, onto a build platform. Common materials used in FDM include acrylonitrile butadiene styrene (ABS), acrylonitrile styrene acrylate (ASA), polylactic acid (PLA), polyamide (PA), and glycol-modified polyethylene terephthalate (PETG) [13,32]. Thermoplastic materials and composites have garnered significant attention due to their favorable mechanical properties and broad applicability across various industries. Notable examples include Onyx thermoplastic materials, which combine nylon with chopped carbon fibers to enhance strength and durability, making them particularly suitable for high-performance drone components [33]. Glass fiber-reinforced polypropylene (PP) composites exhibit excellent stiffness and strength, making them ideal for automotive and industrial equipment applications. Additionally, high-density polyethylene (HDPE) recycled through FDM presents a sustainable solution for marine and industrial applications [34]. Ghabezi et al. [35] further highlighted the circular economy potential by utilizing industrial waste polypropylene and carbon fibers in 3D printing, enabling the production of robust and environmentally friendly products. These examples underscore the opportunities for innovation and sustainability in manufacturing processes across various sectors. Each of these materials offers distinct properties, making the selection of the appropriate material crucial for meeting specific mechanical requirements in various applications.

Understanding the influence of manufacturing parameters and environmental factors on the mechanical properties of FDM-printed materials is essential for ensuring their reliability and performance in practical applications. Factors such as print settings, material properties, and external conditions play a significant role in determining the quality and mechanical properties of the printed objects. In particular, the impact of infill density, printing speed, layer height, and environmental conditions such as temperature and humidity on the final product must be thoroughly investigated to optimize the FDM process [36].

Extensive research has been conducted on the mechanical properties of polymeric and composite materials, including PLA and PLA reinforced with carbon fibers (PLA+CF). PLA is a preferred material for FDM due to its biodegradability, low cost, and ease of printing [37,38]. It is recognized for its excellent printability and dimensional accuracy, making it suitable for a variety of applications, from prototyping to functional parts. Despite its lower tensile strength compared to other engineering-grade materials [39,40], PLA offers notable stiffness and rigidity, which are essential for maintaining structural integrity. Studies have extensively examined the mechanical properties of PLA, including tensile strength, flexural strength, elastic modulus, shear stress, and impact strength [37,41,42,43]. PLA’s high impact resistance and low melting point enhance its versatility across different 3D printers [37,44,45].

The structural parameters of FDM 3D-printed PLA materials, such as infill density and pattern, significantly influence the final properties of the printed products. Higher infill densities increase strength and weight but also result in greater material usage and longer print times, whereas lower densities save material and time at the cost of reduced strength [46]. The choice of infill pattern, such as grid, honeycomb, or triangle, also affects the balance of strength, flexibility, and material usage [47].

The printing environment, including ambient temperature, humidity, and airflow, plays a critical role in the quality of prints. Maintaining a controlled environment with consistent temperature and low humidity is ideal for minimizing issues like warping and cracking during PLA printing [48]. Proper temperature control during the printing process helps achieve uniform mechanical properties and dimensional accuracy. PLA’s hygroscopic nature makes it susceptible to moisture absorption, which can lead to problems such as bubbling, poor layer adhesion, and reduced mechanical strength due to the hydrolysis of polymer chains [49]. Therefore, it is crucial to store PLA filaments in a dry environment and use filament dryers when necessary to maintain optimal print quality. Using an enclosed printing chamber can provide a controlled environment, mitigating the effects of external temperature fluctuations and drafts, which is particularly important for large or complex PLA parts [6].

PLA reinforced with carbon fibers (PLA+CF) exhibits enhanced mechanical properties, including increased strength, stiffness, and thermal stability. Carbon fibers, known for their high tensile strength and exceptional stiffness-to-weight ratio, significantly improve the performance of PLA. PLA+CF composites demonstrate higher tensile strength and better resistance to deformation, making them suitable for demanding applications [50,51,52]. The influence of print settings, material properties, and carbon fiber characteristics on the final properties of PLA+CF components is critical for achieving optimal performance.

The structural parameters of FDM 3D-printed PLA+CF materials are influenced by various factors, including print settings, material properties, and the nature of the carbon fibers. These parameters significantly impact the final properties of the printed components. Infill density, the amount of material used to fill the interior of the print, is crucial for determining the strength and weight of PLA+CF components. Higher infill densities result in stronger and heavier parts, while lower infill densities save material and time but at the expense of strength. The infill pattern, such as honeycomb, grid, or triangular structures, also influences the mechanical performance. Complex patterns like honeycomb can provide a good balance between strength and material usage [47].

The printing environment, including ambient temperature and humidity, plays a significant role in the quality of PLA+CF prints. A controlled environment with consistent temperature and low humidity is ideal for minimizing warping and other printing defects. Variations in environmental conditions can negatively impact the dimensional accuracy and mechanical properties of the printed parts [48]. PLA+CF filaments are hygroscopic, meaning they absorb moisture from the air. High humidity levels can lead to the absorption of water by the PLA+CF filament, causing issues such as bubbling, poor layer adhesion, and reduced mechanical strength due to the hydrolysis of the polymer chains. It is essential to store PLA+CF filaments in a dry environment and use filament dryers if necessary to maintain optimal print quality [49].

Airflow around the print area can influence the cooling rate of the printed material. Uncontrolled airflow, such as drafts from open windows or fans, can cause uneven cooling, leading to warping, layer separation, and surface defects. Controlled airflow and proper ventilation within an enclosed printing chamber can help achieve consistent cooling rates and improved print quality [53].

The study in [54] investigated the effects of temperature and humidity on carbon-fiber-reinforced plastic (CFRP) composites produced using AM. Samples were exposed to warm and wet, warm and dry, and cold and dry conditions, comparing their mechanical performance to those tested immediately post-fabrication. The results indicated minimal impact from warm temperatures, whereas near-zero cold temperatures significantly affected the materials after 96 and 250 h. Temperature was identified as a more influential factor than humidity.

A critical factor affecting the mechanical properties of polymeric materials and composites is their microstructure. Defects such as microcracks and porosity can greatly influence the strength, stiffness, ductility, and durability of these materials [13]. Understanding how cooling lubricants affect these microstructural defects is essential for advancing material development and improving their mechanical properties. Despite extensive literature reviews, no comprehensive study has examined the impact of cooling lubricants on the mechanical properties of FDM 3D-printed polymer materials and composites, highlighting the need for this research.

Cooling lubricants used in machining consist of complex chemicals that interact differently under various conditions. Polymeric materials and their composites can absorb these components, influenced by both the chemical composition and environmental conditions like temperature and pressure. This absorption can alter microstructural properties, leading to microcracks and defects by weakening polymer chain bonds, resulting in reduced strength and increased brittleness. Additionally, chemical interactions between the lubricant and the material can further degrade the microstructure, significantly impacting mechanical properties and reducing strength, elasticity, and ductility. This has critical implications for the safety, durability, and performance of final products in various industries.

Building on these findings, our study examines the impact of infill density and prolonged exposure to cooling lubricants (Zubora 77 H Ultra, Zeller+Gmelin, Eislingen, Germany) on the mechanical properties of PLA and PLA+CF samples. Zubora 77 H Ultra, a water-miscible cooling lubricant, excels in machining and metalworking by providing exceptional lubricity, reducing friction and wear, and enhancing tool life and surface finish. Its efficient heat removal prevents thermal damage, while its stable emulsion ensures consistent performance. The lubricant also offers robust corrosion protection and biostability, reducing maintenance needs, and is environmentally friendly, free from harmful substances like nitrites, phenols, and chlorine.

By systematically studying these materials under controlled conditions, we gained insights into their response to cooling lubricant exposure, focusing on mechanical parameters such as maximum force, modulus of elasticity (Young’s modulus), tensile strength, and strain. These properties are critical for assessing structural integrity and performance under various loading conditions. Evaluating changes before and after lubricant exposure allows us to gauge material degradation, mechanical performance loss, and differences between pure and reinforced materials. The primary mechanism involves microstructural defects induced by absorbed lubricant molecules, which significantly alter mechanical properties.

Our research expands the understanding of cooling lubricants’ influence on polymeric and composite materials, aiding in the selection of appropriate materials for environments exposed to lubricants. This enables engineers and designers to optimize component performance and reliability. Understanding the impact on mechanical parameters like modulus of elasticity, maximum force, tensile strength, and strain helps predict material resistance to degradation and mechanical performance loss, crucial for ensuring safety and durability in industrial applications exposed to cooling lubricants.

## 2. Materials and Methods

### 2.1. PLA and PLA+CF Materials Specification and Mechanical Parameters

The specifications and mechanical parameters of the PLA and PLA+CF filaments used in this study, as provided by the manufacturer [55], are detailed in Table 1 and Table 2. The mechanical parameters presented in Table 2 are sourced from the filament manufacturer [55] and correspond to specimens with 100% infill density.

Based on the material specifications supplied by the filament manufacturer [55], this study utilized filaments with a circular cross-section, an average diameter of 1.75 mm, and minimal standard deviation. This study focuses on short carbon fibers. The optimal percentage of carbon fibers to enhance the mechanical properties of CF-PLA objects is typically around 10–20%. The addition of carbon fibers can increase mechanical strength by 20–50%, depending on the composition and manufacturing techniques used [56,57]. Continuous carbon fibers could potentially offer even greater improvements in strength and stiffness; however, they were not the subject of this study.

**Table 2:** Mechanical parameters of PLA and PLA+CF filaments [55].

Parameters	Test Method	Material Type	
		PLA	PLA+CF
Density (g/cm^3^)	ISO 1183 [58]	1.24	~1.29
Young’s modulus (MPa)	ISO 527 [59]	1000–1100	1100–1300
Tensile strength (MPa)	ISO 527	45–49	40–45
Elongation at break (%)	ISO 527	13.5–15.5	11.5–13.5
Heat deflection temperature (°C)	ISO 75 [60]	53	60

### 2.2. Preparation and 3D Printing of Tensile Test Specimens

The experimental study involved using the 3D CAD software *SolidWorks 2020* for the specimen design, slicer *Bambu Studio* software (v01.09.03.50) for adjusting the printer parameters [61], and the *Bambu Lab X1 Carbon Combo* 3D printer [62] for manufacturing the tensile-test specimens.

**Figure 1:**
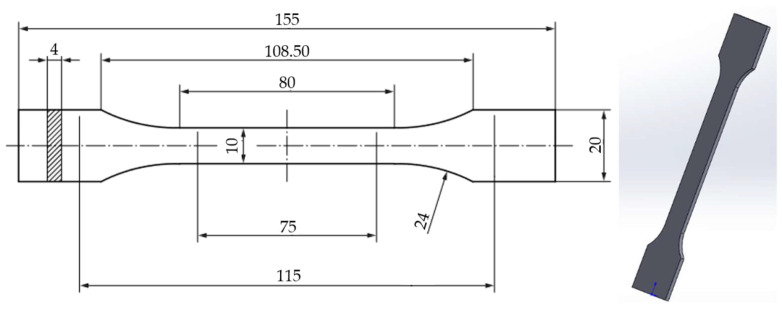
3D model of tensile-test specimens according to the ISO 527-2-2012 standard [36].

The tensile test specimen model was designed in SolidWorks 2020 3D CAD software according to the ISO 527-2 standard [59] (Figure 1). The model was then converted to STL format for use in the slicer software.

The STL file was used as the input parameter for setting and adjusting the process parameters for 3D printing.

The settings and adjusting of the process parameter for 3D printing were configured using the *Bambu Studio* slicer software for the FDM 3D printer. The main printing parameters for the PLA and PLA+CF materials are provided in Table 3.

**Figure 2:**
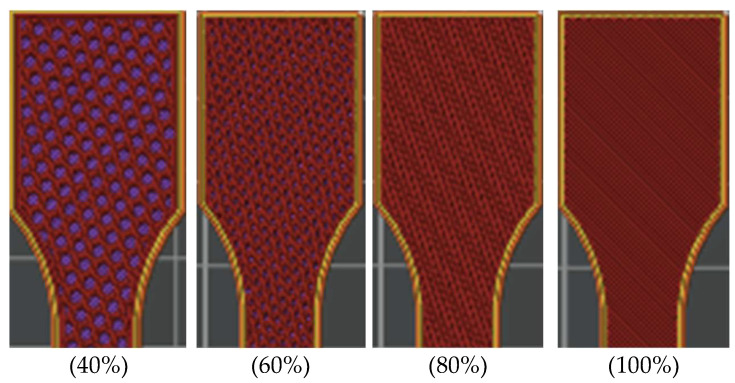
Tensile-test specimens with “*Honeycomb*” infill pattern and 40%, 60%, 80%, and 100% infill density.

In the slicer *Bambu Studio* software (Bambu Studio v.1.7.7.89.), users can choose from different infill patterns: concentric, rectilinear, grid, line, triangles, tri-hexagon, honeycomb, monotonic, etc. For this study, the “*Honeycomb*” infill pattern with a 40%, 60%, 80% and 100% fill density was used for all tensile and compressive test specimens (Figure 2). The top surface pattern, bottom surface pattern, and internal solid infill were all created using a “*Monotonic*” infill pattern.

Once all the parameters were set in the slicer *Bambu Studio* software (Table 3), the *G-code* was generated and sent to the computer managing the 3D printer. Following this, the *Bambu Lab X1 Carbon Combo* 3D printer was used to print all the tensile-test specimens.

For both materials, 60 identical tensile test specimens were printed, resulting in a total of 120 specimens. All the tensile-test specimens were produced using 1000 g spools of PLA and PLA+CF materials. The PLA+CF composite material was prepared by the filament manufacturer [55].

### 2.3. Tensile Testing for 3D-Printed Specimens

The input for the tensile testing is represented by the 3D-printed tensile-test specimens. The 3D-printed tensile-test specimens of both materials were categorized into three groups and subjected to testing at specific time intervals.

Initially, 20 samples of each material (different infill density) are tested without any exposure to environmental factors. Subsequently, the remaining samples are immersed in a container filled with cooling lubricant (*Zubora 77 H Ultra*). The second group of samples is tested after 7 days, while the final group of samples for both materials is tested after a 30-day period. The samples were exposed to cooling lubricants for 7 and 30 days to determine the short-term and mid-term effects on mechanical properties. Longer periods were not included due to the time constraints of the study.

For each group, four sets of specimens (Case) were prepared, each with 5 samples, corresponding to different infill densities (40%, 60%, 80%, and 100%).

Tensile tests were conducted using the *Zwick Z 600 universal testing machine* (Zwick-Roell, Ulm, Germany) with a maximum load capacity of 250 kN. The tensile tests were performed according to the ISO 527-2 standard [59]. All the 3D-printed tensile-test specimens were statically loaded.

The tensile-test data acquisition and monitoring were conducted using *testXpert II* (V3.6) software (Zwick-Roell, Ulm, Germany). This advanced software enables the collection of data such as displacement (mm), force (N), strength (MPa), strain (%), Young’s modulus (MPa), etc., for each test, as well as the generation of strength–strain curves.

After acquiring the tensile-test data, the results were processed using Excel and are presented in the following section.

## 3. Results

### 3.1. Tensile Properties of the FDM 3D-Printed PLA Specimens

The tensile test data for all FDM 3D-printed PLA specimens are organized into three distinct groups: (1) data pertaining to tensile-tested 3D-printed PLA specimens that were not exposed to cooling lubricant (Section 3.1.1), (2) data concerning tensile-tested 3D-printed PLA specimens that were exposed to cooling lubricant for 7 days (Section 3.1.2), and (3) data relating to tensile-tested 3D-printed PLA specimens that were exposed to cooling lubricant for 30 days (Section 3.1.3).

#### 3.1.1. Tensile Properties of FDM 3D-Printed PLA Specimens Not Exposed to Cooling Lubricant

Table 4 presents the tensile test data for FDM 3D-printed PLA specimens that were not exposed to cooling lubricant. The data from the tensile tests are categorized into four distinct sections: (1) information from FDM 3D-printed PLA samples made with PLA filament and 40% infill density (PLA_4T1, PLA_4T2, PLA_4T3, PLA_4T4, and PLA_4T5)—referred to as Case 1, (2) details of FDM 3D-printed PLA samples manufactured with PLA filament and 60% infill density (PLA_6T1, PLA_6T2, PLA_6T3, PLA_6T4, and PLA_6T5)—known as Case 2, (3) data from FDM 3D-printed PLA samples created using PLA filament with 80% infill density (PLA_8T1, PLA_8T2, PLA_8T3, PLA_8T4, and PLA_8T5)—termed Case 3, and (4) information on FDM 3D-printed PLA samples made from PLA filament with a 100% infill density (PLA_1T1, PLA_1T2, PLA_1T3, PLA_1T4, and PLA_1T5)—designated as Case 4.

**Figure 3:**
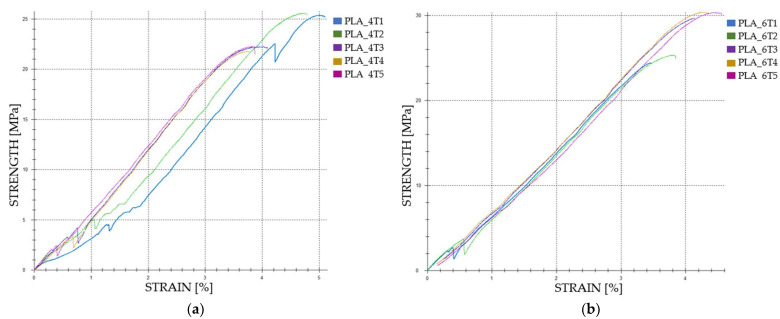

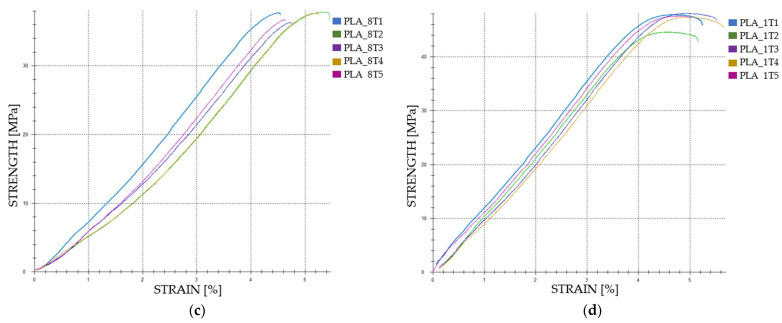
The 3D printed PLA specimens tested: the strength-strain curves: (**a**) PLA tensile-tested specimens with 40% fill density—Case 1; (**b**) PLA tensile-tested specimens with 60% fill density—Case 2, (**c**) PLA tensile-tested specimens with 80% fill density—Case 3, and (**d**) PLA tensile-tested specimens with 100% fill density—Case 4. All the PLA tensile-tested specimens were not exposed to cooling lubricant.

The graphical representations of the strength and strain for individual FDM 3D-printed PLA tensile-tested specimens that were not exposed to cooling lubricant are shown in Figure 3.

When comparing Case 1 and Case 2, tensile strength increases from 22.49 MPa to 26.95 MPa, a 19.85% rise. Case 3 sees tensile strength reaching 35.54 MPa, marking a 58.06% increase over Case 1. In Case 4, tensile strength peaks at 45.00 MPa, a 100.09% increase from Case 1. When comparing Case 2 to Case 3, tensile strength increases by 31.86%, and from Case 2 to Case 4, it increases by 66.92%. The increase from Case 3 to Case 4 is 26.55%.

Strain values exhibit more variability. Between Case 1 and Case 2, strain decreases slightly from 4.23% to 3.98%, a 5.91% reduction. However, strain increases to 4.80% in Case 3, representing a 13.49% rise over Case 1, and to 4.68% in Case 4, a 10.64% increase from Case 1. Comparing Case 2 to Case 3, strain increases by 20.60%, while the increase from Case 2 to Case 4 is 17.59%. Notably, there is a slight decrease in strain from Case 3 to Case 4 by 2.50%.

**Figure 4:**
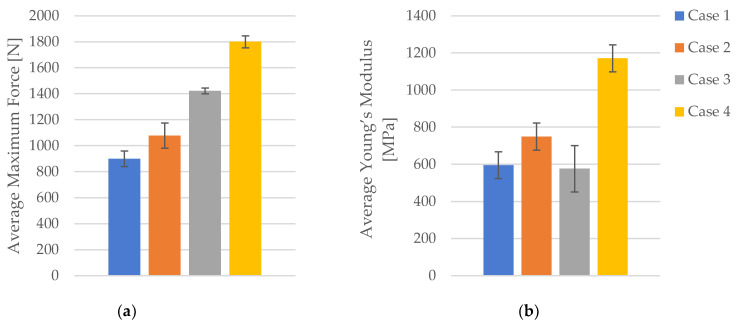
The mechanical parameters of FDM 3D-printed PLA specimens tested that were not exposed to cooling lubricant: (**a**) average maximum force, (**b**) average Young’s modulus.

Average values of maximal force and Young’s modulus are presented in Figure 4.

The comparative analysis indicates that increasing the infill density generally enhances the mechanical properties of FDM 3D-printed PLA specimens. The maximum force and tensile strength consistently increased with higher infill densities, showing significant improvements. While the strain values showed some variability, the overall trend suggests a slight increase with higher densities. Young’s modulus demonstrated a substantial increase, particularly from 40% to 100% infill density, indicating greater stiffness and rigidity in specimens with higher infill.

#### 3.1.2. Tensile Properties of FDM 3D-Printed PLA Specimens Exposed to Cooling Lubricant for 7 Days

The tensile test data for the FDM 3D-printed PLA specimens that were exposed to cooling lubricant for 7 days are shown in Table 5.

The tensile-test data are divided into four distinct categories: (1) FDM 3D-printed PLA samples with 40% infill density (PLA_4T71, PLA_4T72, PLA_4T73, PLA_4T74, and PLA_4T75)—referred to as Case 5, (2) FDM 3D-printed PLA samples with 60% infill density (PLA_6T71, PLA_6T72, PLA_6T73, PLA_6T74, and PLA_6T75)—designated as Case 6, (3) FDM 3D-printed PLA samples with 80% infill density (PLA_8T71, PLA_8T72, PLA_8T73, PLA_8T74, and PLA_8T75)—referred to as Case 7, and (4) FDM 3D-printed PLA samples with 100% infill density (PLA_1T71, PLA_1T72, PLA_1T73, PLA_1T74, and PLA_1T75)—designated as Case 8.

**Figure 5:**
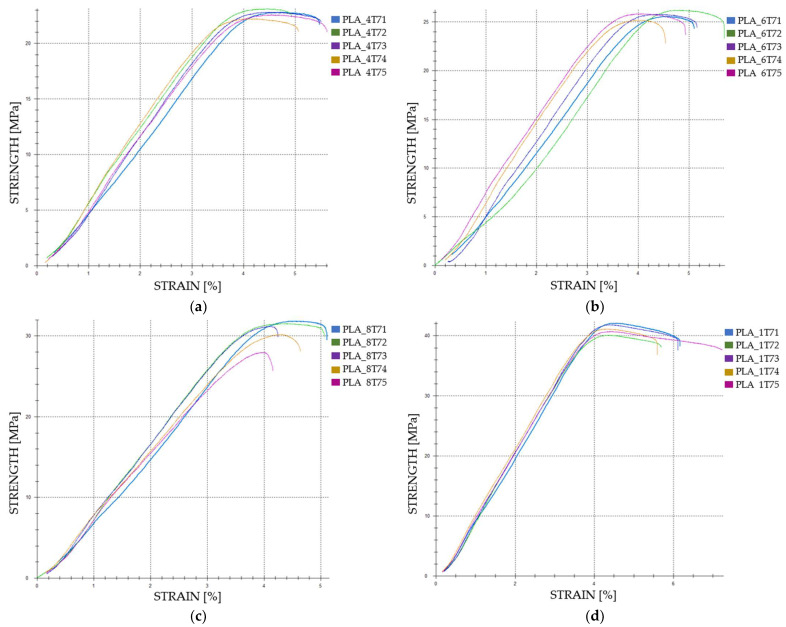
The 3D printed PLA specimens tested: the strength-strain curves: (**a**) PLA tensile-tested specimens with 40% fill density—Case 5; (**b**) PLA tensile-tested specimens with 60% fill density—Case 6, (**c**) PLA tensile-tested specimens with 80% fill density—Case 7, and (**d**) PLA tensile-tested specimens with 100% fill density—Case 8. All PLA tensile-tested specimens were exposed to cooling lubricant for 7 days.

The graphical representations of the tensile strength and strain for individual FDM 3D-printed PLA tensile-tested specimens that were exposed to cooling lubricant for 7 days are shown in Figure 5.

The tensile properties of FDM 3D-printed PLA specimens exposed to cooling lubricant for seven days reveal significant differences based on varying infill densities.

In Case 5, the average tensile strength is 21.78 MPa. This increases to 24.69 MPa in Case 6, reflecting a 13.21% rise. Further enhancement is observed in Case 7, where tensile strength reaches 29.32 MPa, a 35.34% increase over Case 5. The highest value is in Case 8, at 39.47 MPa, marking an 82.88% increase from Case 5. Comparing Case 6 to Case 7, the tensile strength increases by 19.56%, and from Case 6 to Case 8, it rises by 61.51%. The increase from Case 7 to Case 8 is 35.02%.

Strain values exhibit minor fluctuations across the different infill densities. In Case 5, the average strain is 4.29%. This slightly decreases to 4.17% in Case 6, a 2.80% reduction. In Case 7, the strain remains relatively stable at 4.16%, showing a 3.03% decrease from Case 5. However, in Case 8, the strain increases marginally to 4.26%, indicating a 0.70% decrease from Case 5. Comparing Case 6 to Case 7, there is a minimal decrease of 0.24%, while Case 6 to Case 8 shows a 2.16% increase. The strain from Case 7 to Case 8 increases by 2.40%.

**Figure 6:**
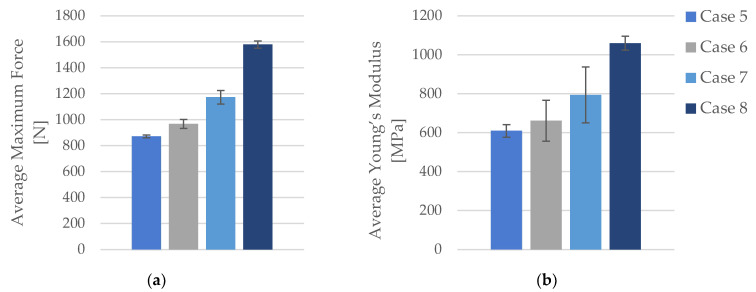
The mechanical parameters of FDM 3D-printed PLA specimens tested that were exposed to cooling lubricant for 7 days: (**a**) average maximum force, (**b**) average Young’s modulus.

In terms of maximum force, there is a noticeable progression as the infill density increases (Figure 6a). For Case 5, the average maximum force is 871.05 N. This value increases to 967.64 N in Case 6, marking a 13.13% rise. The trend continues in Case 7, where the maximum force reaches 1172.84 N, a 34.39% increase over Case 5. The most substantial increase is observed in Case 8, where the maximum force jumps to 1578.81 N, representing an 81.00% increase from Case 5. Comparatively, Case 6 to Case 7 sees an 18.79% rise, while Case 6 to Case 8 shows a 60.00% increase. Finally, the increase from Case 7 to Case 8 is 34.63%.

Young’s modulus shows considerable improvement with increasing infill density (Figure 6b). The average Young’s modulus for Case 5 is 609.2 MPa. This value rises to 661.40 MPa in Case 6, representing an 8.56% increase. In Case 7, Young’s modulus reaches 794.40 MPa, marking a 30.40% rise over Case 5. The highest value is observed in Case 8, at 1059.20 MPa, which is a 73.83% increase from Case 5. When comparing Case 6 to Case 7, Young’s modulus increases by 20.11%, and from Case 6 to Case 8, it rises by 60.10%. The increase from Case 7 to Case 8 is 33.30%.

#### 3.1.3. Tensile Properties of FDM 3D-Printed PLA Specimens Exposed to Cooling Lubricant for 30 Days

The tensile-test data for the FDM 3D-printed PLA specimens that were exposed to cooling lubricant for 30 days are shown in Table 6.

The tensile-test data are divided into four distinct categories: (1) FDM 3D-printed PLA samples with 40% infill density (PLA_4T301, PLA_4T302, PLA_4T303, PLA_4T304, and PLA_4T305)—referred to as Case 9, (2) FDM 3D-printed PLA samples with 60% infill density (PLA_6T301, PLA_6T302, PLA_6T303, PLA_6T304, and PLA_6T305)—designated as Case 10, (3) FDM 3D-printed PLA samples with 80% infill density (PLA_8T301, PLA_8T302, PLA_8T303, PLA_8T304, and PLA_8T305)—referred to as Case 11, and (4) FDM 3D-printed PLA samples with 100% infill density (PLA_1T301, PLA_1T302, PLA_1T303, PLA_1T304, and PLA_1T305)—designated as Case 12.

The tensile-test data for FDM 3D-printed PLA specimens exposed to cooling lubricant for a duration of 30 days reveal significant alterations in their mechanical properties, as presented in Table 6.

**Figure 7:**
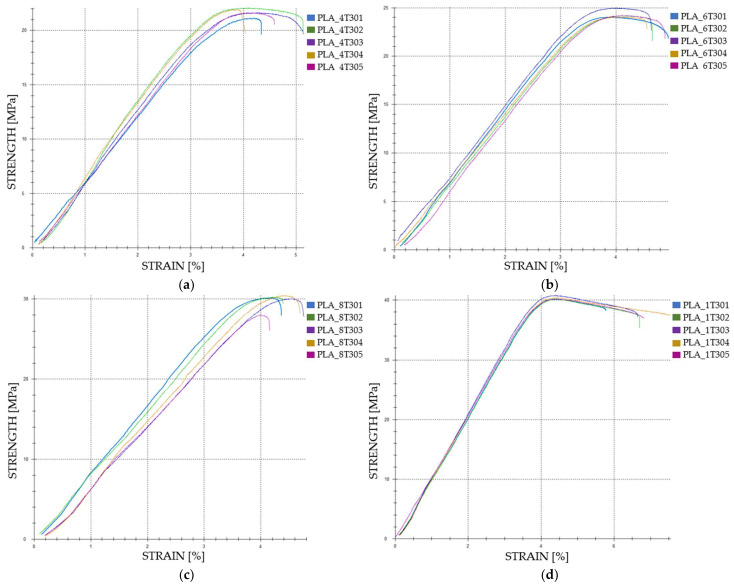
The 3D printed PLA specimens tested: the strength-strain curves: (**a**) PLA tensile-tested specimens with 40% fill density—Case 9; (**b**) PLA tensile-tested specimens with 60% fill density—Case 10, (**c**) PLA tensile-tested specimens with 80% fill density—Case 11, and (**d**) PLA tensile-tested specimens with 100% fill density—Case 12. All the PLA tensile-tested specimens were exposed to cooling lubricant for 30 days.

Tensile strength shows marked improvements with increasing infill density. The average tensile strength for 40% infill density was 20.84 MPa (Case 9, Figure 7a). At 60% infill density (Case 10), it increased to 23.35 MPa, a 12.04% rise (Figure 7b). Further enhancement to 80% infill density (Case 11) resulted in an average tensile strength of 28.56 MPa, a 37.06% increase over 40% infill density (Figure 7c). The highest tensile strength was observed at 100% infill density (Case 12), with an average value of 38.65 MPa, marking an 85.45% increase compared to Case 9. These results demonstrate that greater infill densities significantly enhance tensile strength, which is critical for the mechanical performance of the specimens.

Strain exhibited less pronounced changes compared to maximum force and tensile strength. The average strain for specimens with a 40% infill density was 3.99% (Case 9). At 60% infill density (Case 10), the average strain slightly decreased to 3.96%. However, increasing the infill density to 80% (Case 11) resulted in an average strain of 4.14%, a 3.76% increase compared to the 40% infill density. At 100% infill density (Case 12), the average strain reached 4.32% (Figure 7d), an 8.28% increase compared to Case 9. Although the changes in strain are less dramatic, they still show a positive trend with increasing infill density.

**Figure 8:**
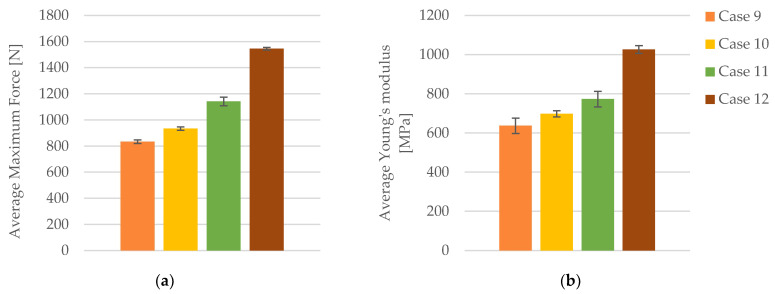
The mechanical parameters of FDM 3D-printed PLA specimens tested that were exposed to cooling lubricant for 30 days: (**a**) average maximum force, (**b**) average Young’s modulus.

In terms of maximum force, a clear trend of increasing values with higher infill densities is observed. Specimens with a 40% infill density (Case 9) exhibited an average maximum force of 833.71 N (Figure 8a). Increasing the infill density to 60% (Case 10) resulted in an average maximum force of 933.93 N, representing a 12.01% increase compared to Case 9. Further increasing the infill density to 80% (Case 11) led to an average maximum force of 1142.47 N, a 37.28% increase over the 40% infill density. The most significant enhancement was seen with a 100% infill density (Case 12), where the average maximum force reached 1545.90 N, an impressive 85.38% increase compared to Case 9. These data clearly demonstrate that higher infill densities substantially augment the maximum force that the specimens can withstand.

Young’s modulus, a measure of material stiffness, showed significant improvements with higher infill densities. The average Young’s modulus for 40% infill density was 637 MPa (Case 9, Figure 8b). At 60% infill density (Case 10), the Young’s modulus increased to 698.40 MPa, representing a 9.64% rise. Further increasing the infill density to 80% (Case 11) resulted in an average Young’s modulus of 773.20 MPa, a 21.39% increase over the 40% infill density. The highest Young’s modulus was recorded at 100% infill density (Case 12), with an average value of 1026.20 MPa, indicating a 61.13% increase compared to Case 9. These findings indicate that higher infill densities significantly enhance the stiffness of the specimens.

In conclusion, this analysis unequivocally demonstrates that increasing the infill density of FDM 3D-printed PLA specimens, even after 30 days of exposure to cooling lubricant, significantly enhances their mechanical properties. Higher infill densities lead to higher values of maximum force, tensile strength, and Young’s modulus, which indicate superior material performance. The strain also exhibits a slight increase, suggesting improved elasticity of the specimens.

**Figure 9:**
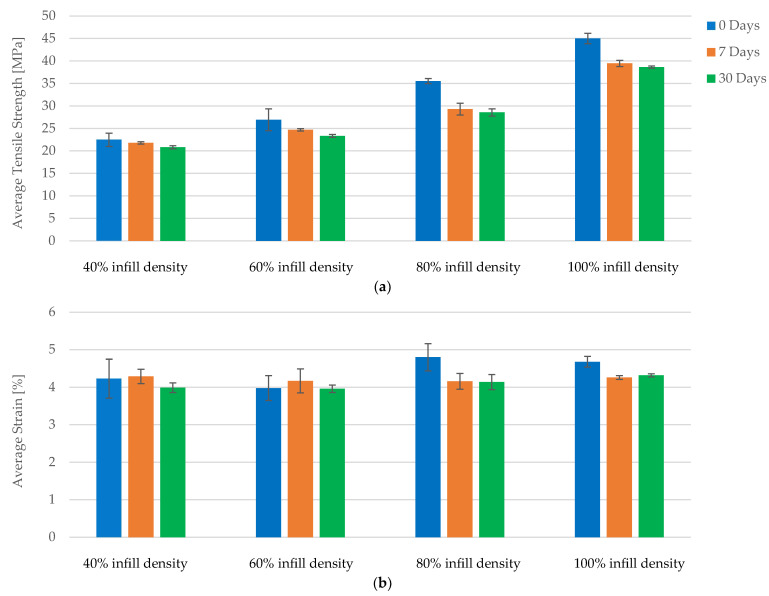
The mechanical parameters of FDM 3D-printed PLA specimens tested: (**a**) average tensile strength, (**b**) average strain.

A comparative analysis of the tensile properties of FDM 3D-printed PLA specimens reveals that increased infill density significantly enhances mechanical performance. Across all conditions—no exposure, 7 days of exposure, and 30 days of exposure to cooling lubricant—the data consistently demonstrate improvements in maximum force and tensile strength with higher infill densities. Specimens with 100% infill density exhibit up to an 85.45% increase in tensile strength compared to those with 40% infill density (Figure 9a), underscoring the critical role of infill density in enhancing structural integrity and load-bearing capacity.

Strain values exhibit minor fluctuations across different infill densities, indicating stable elasticity. While higher infill densities enhance mechanical properties, the impact on elasticity is less pronounced, which is beneficial for applications requiring both strength and flexibility (Figure 9b).

Young’s modulus shows significant improvements with higher infill densities, indicating increased rigidity and resistance to deformation. Across all conditions, the increase in Young’s modulus from 40% to 100% infill density ranges from 61.13% to 76.60%, illustrating that higher infill densities not only improve strength but also contribute to overall material stiffness, making it suitable for applications where rigidity is essential.

Exposure to cooling lubricant for 7 and 30 days does not significantly alter the positive effects of increased infill density on mechanical properties. The improvements in maximum force, tensile strength, and Young’s modulus with higher infill densities remain consistent, indicating that the benefits of increased infill are retained even under potentially degrading conditions.

### 3.2. Tensile Properties of the FDM 3D-Printed PLA+CF Specimens

The tensile-test data for all FDM 3D-printed PLA+CF specimens are organized into three distinct groups: (1) data pertaining to tensile-tested 3D-printed PLA+CF specimens that were not exposed to cooling lubricant (Section 3.2.1), (2) data pertaining to tensile-tested 3D-printed PLA+CF specimens exposed to cooling lubricant for a duration of 7 days (Section 3.2.2), and (3) data associated with tensile-tested 3D-printed PLA+CF specimens exposed to cooling lubricant for a period of 30 days (Section 3.2.3).

#### 3.2.1. Tensile Properties of the FDM 3D-Printed PLA+CF Specimens Not Exposed to Cooling Lubricant

The tensile-test data for the FDM 3D-printed PLA+CF specimens that were not exposed to cooling lubricant are shown in Table 7.

The tensile-test data are classified into four distinct categories: (1) FDM 3D-printed PLA+CF samples with 40% infill density (PLA+CF_4T1, PLA+CF_4T2, PLA+CF_4T3, PLA+CF_4T4, and PLA+CF_4T5), referred to as Case 13; (2) FDM 3D-printed PLA+CF samples with 60% infill density (PLA+CF_6T1, PLA+CF_6T2, PLA+CF_6T3, PLA+CF_6T4, and PLA+CF_6T5), designated as Case 14; (3) FDM 3D-printed PLA+CF samples with 80% infill density (PLA+CF_8T1, PLA+CF_8T2, PLA+CF_8T3, PLA+CF_8T4, and PLA+CF_8T5), referred to as Case 15; and (4) FDM 3D-printed PLA+CF samples with 100% infill density (PLA+CF_1T1, PLA+CF_1T2, PLA+CF_1T3, PLA+CF_1T4, and PLA+CF_1T5), designated as Case 16.

In Case 13, with an infill density of 40%, the average tensile strength is recorded at 23.09 MPa. This value increases to 27.49 MPa in Case 14, representing a 19.05% increase. Further enhancement is observed in Case 15, where the tensile strength reaches 34.74 MPa, marking a 50.46% increase compared to Case 13. The highest value is observed in Case 16, with a tensile strength of 42.54 MPa, signifying an 84.27% increase from Case 13. When comparing Case 14 to Case 15, the tensile strength increases by 26.39%, while the increase from Case 14 to Case 16 is 54.72%. The increase from Case 15 to Case 16 is 22.44%.

The strain values exhibit minor fluctuations across the different infill densities. In Case 13, the average strain is 3.98%. This slightly increases to 4.06% in Case 14, representing a 2.01% increase. In Case 15, the strain remains relatively stable at 4.24%, indicating a 6.53% increase compared to Case 13. In Case 16, the strain marginally rises to 4.33%, demonstrating an 8.79% increase relative to Case 13. When comparing Case 14 to Case 15, a minimal increase of 4.43% is observed, while the increase from Case 14 to Case 16 is 6.65%. The increase from Case 15 to Case 16 is 2.12%.

**Figure 10:**
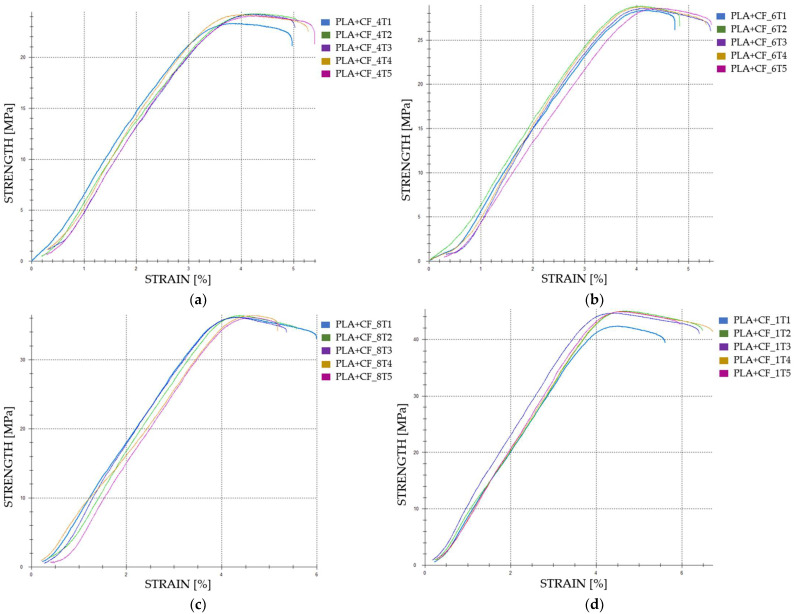
The 3D printed PLA+CF specimens tested: the strength-strain curves: (**a**) PLA+CF tensile-tested specimens with 40% fill density—Case 13; (**b**) PLA+CF tensile-tested specimens with 60% fill density—Case 14, (**c**) PLA+CF tensile-tested specimens with 80% fill density—Case 15, and (**d**) PLA+CF tensile-tested specimens with 100% fill density—Case 16. All the PLA+CF tensile-tested specimens were not exposed to cooling lubricant.

The graphical representations of the strength and strain for individual FDM 3D-printed PLA+CF tensile-tested specimens that were not exposed to cooling lubricant are shown in Figure 10.

**Figure 11:**
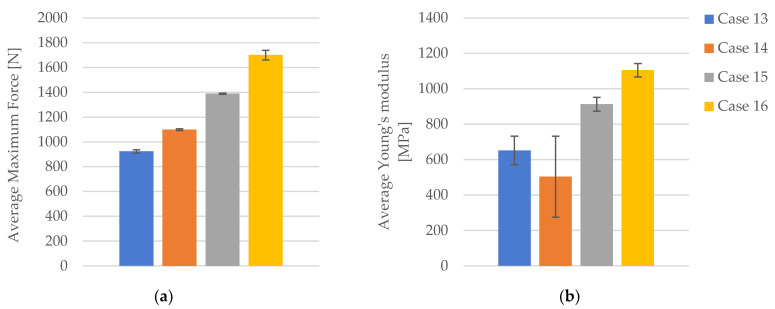
The mechanical parameters of FDM 3D-printed PLA+CF specimens tested that were not exposed to cooling lubricant: (**a**) average maximum force, (**b**) average Young’s modulus.

The maximum force increases significantly with higher infill densities. In Case 13, the average maximum force is 923.76 N (Figure 11a). This value rises to 1099.63 N in Case 14, representing a 19.05% increase. In Case 15, the maximum force reaches 1389.59 N, which is a 50.46% increase compared to Case 13. The highest value is observed in Case 16, where the maximum force is 1701.51 N (Figure 11a), marking an 84.27% increase relative to Case 13. Comparing Case 14 to Case 15, the maximum force shows a 26.39% increase, while the increase from Case 14 to Case 16 is 54.72%. The increase from Case 15 to Case 16 is 22.44%.

The stiffness of the material, as indicated by Young’s modulus, increases significantly with higher infill densities. In Case 13, the average Young’s modulus is 652 MPa (Figure 11b). This value decreases to 504.20 MPa in Case 14, representing a 22.65% reduction. In Case 15, Young’s modulus increases to 913 MPa, marking a 40.03% increase compared to Case 13. The highest value is observed in Case 16, where Young’s modulus is 1105.80 MPa, signifying a 69.56% increase relative to Case 13. When comparing Case 14 to Case 15, an 81.06% increase is noted, while the increase from Case 14 to Case 16 is 119.32%. The increase from Case 15 to Case 16 is 21.17%.

#### 3.2.2. Tensile Properties of the FDM 3D-Printed PLA+CF Specimens Exposed to Cooling Lubricant for 7 Days

The tensile-test data for the FDM 3D-printed PLA+CF specimens that were exposed to cooling lubricant for a duration of 7 days are shown in Table 8.

The tensile-test data are categorized into four distinct groups: (1) FDM 3D-printed PLA+CF samples with 40% infill density (PLA+CF_4T71, PLA+CF_4T72, PLA+CF_4T73, PLA+CF_4T74, and PLA+CF_4T75), referred to as Case 17; (2) FDM 3D-printed PLA+CF samples with 60% infill density (PLA+CF_6T71, PLA+CF_6T72, PLA+CF_6T73, PLA+CF_6T74, and PLA+CF_6T75), designated as Case 18; (3) FDM 3D-printed PLA+CF samples with 80% infill density (PLA+CF_8T71, PLA+CF_8T72, PLA+CF_8T73, PLA+CF_8T74, and PLA+CF_8T75), referred to as Case 19; and (4) FDM 3D-printed PLA+CF samples with 100% infill density (PLA+CF_1T71, PLA+CF_1T72, PLA+CF_1T73, PLA+CF_1T74, and PLA+CF_1T75), designated as Case 20.

**Figure 12:**
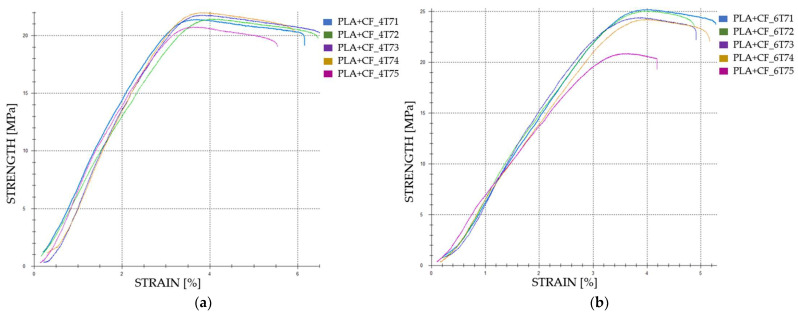

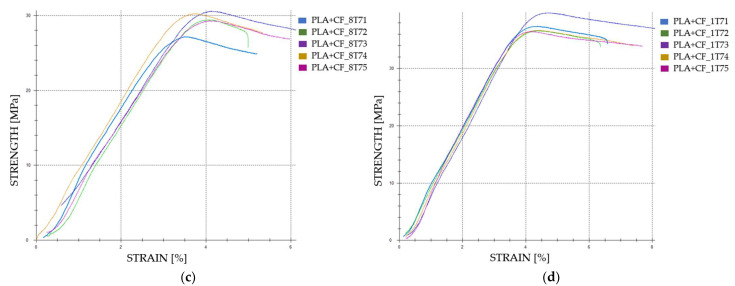
The 3D printed PLA+CF specimens tested: the strength-strain curves: (**a**) PLA+CF tensile-tested specimens with 40% fill density—Case 17; (**b**) PLA+CF tensile-tested specimens with 60% fill density—Case 18, (**c**) PLA+CF tensile-tested specimens with 80% fill density—Case 19, and (**d**) PLA+CF tensile-tested specimens with 100% fill density—Case 20. All the PLA+CF tensile-tested specimens were exposed to cooling lubricant for a period of 7 days.

The graphical representations of the tensile strength and strain for individual FDM 3D-printed PLA+CF tensile-tested specimens that were exposed to cooling lubricant for a period of 7 days are shown in Figure 12.

The tensile strength demonstrates significant improvements with increasing infill density. In Case 17, the average tensile strength is recorded at 20.70 MPa. This value increases to 23.07 MPa in Case 18, representing a 13.37% rise. Further enhancement is observed in Case 19, where the tensile strength reaches 28.25 MPa, indicating a 36.49% increase over Case 17. The highest tensile strength is noted in Case 20, at 35.83 MPa, marking a 73.10% increase from Case 17. When comparing Case 18 to Case 19, the tensile strength increases by 20.37%, and from Case 18 to Case 20, it rises by 52.66%. The increase from Case 19 to Case 20 is 26.85%.

The strain values exhibit minor fluctuations across the different infill densities. In Case 17, the average strain is 3.67%. This value increases slightly to 3.76% in Case 18, reflecting a 2.45% rise. In Case 19, the strain remains relatively stable at 3.79%, representing a 3.27% increase from Case 17. In Case 20, the strain increases marginally to 4.24%, indicating a 15.53% rise from Case 17. Comparing Case 18 to Case 19, there is a minimal increase of 0.80%, while from Case 18 to Case 20, the increase is 12.77%. The strain from Case 19 to Case 20 increases by 11.87%.

**Figure 13:**
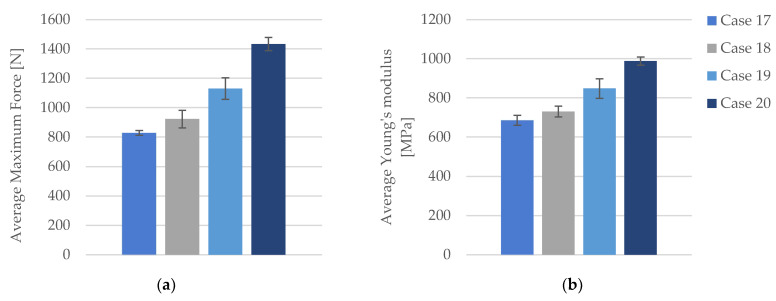
The mechanical parameters of FDM 3D-printed PLA+CF specimens tested that were exposed to cooling lubricant for a period of 7 days: (**a**) average maximum force, (**b**) average Young’s modulus.

The maximum force exhibited by the specimens significantly increases with higher infill densities. In Case 17, with a 40% infill density, the average maximum force is 828.08 N (Figure 13a). This value rises to 922.86 N in Case 18, representing an 11.45% increase. Further enhancement is observed in Case 19, where the maximum force reaches 1130.18 N, indicating a 36.48% increase compared to Case 17. The highest value is noted in Case 20, with a maximum force of 1433.19 N, marking a 73.07% increase from Case 17. When comparing Case 18 to Case 19, the maximum force increases by 22.46%, while the increase from Case 18 to Case 20 is 55.30%. The increase from Case 19 to Case 20 is 26.81%.

The stiffness of the material, as indicated by Young’s modulus, exhibits significant increases with higher infill densities. In Case 17, the average Young’s modulus is 685.80 MPa (Figure 13b). This value rises to 730.20 MPa in Case 18, representing a 6.47% increase. In Case 19, Young’s modulus further increases to 847.80 MPa, marking a 23.62% increase compared to Case 17. The highest value is observed in Case 20, where Young’s modulus reaches 988.00 MPa, signifying a 44.07% increase relative to Case 17. Comparing Case 18 to Case 19, a 16.11% increase is noted, while the increase from Case 18 to Case 20 is 35.31%. The increase from Case 19 to Case 20 is 16.54%.

The quantitative and qualitative analysis of tensile properties for FDM 3D-printed PLA+CF specimens exposed to cooling lubricant for seven days clearly demonstrates that increasing the infill density results in significant improvements in maximum force, tensile strength, and Young’s modulus, with relatively minor fluctuations in strain.

#### 3.2.3. Tensile Properties of the FDM 3D-Printed PLA+CF Specimens Exposed to Cooling Lubricant for 30 Days

The tensile-test data for the FDM 3D-printed PLA+CF specimens that were exposed to cooling lubricant for a period of 30 days are shown in Table 9.

The tensile-test data are categorized into four distinct groups: (1) FDM 3D-printed PLA+CF samples with 40% infill density (PLA+CF_4T301, PLA+CF_4T302, PLA+CF_4T303, PLA+CF_4T304, and PLA+CF_4T305), referred to as Case 21; (2) FDM 3D-printed PLA+CF samples with 60% infill density (PLA+CF_6T301, PLA+CF_6T302, PLA+CF_6T303, PLA+CF_6T304, and PLA+CF_6T305), designated as Case 22; (3) FDM 3D-printed PLA+CF samples with 80% infill density (PLA+CF_8T301, PLA+CF_8T302, PLA+CF_8T303, PLA+CF_8T304, and PLA+CF_8T305), referred to as Case 23; and (4) FDM 3D-printed PLA+CF samples with 100% infill density (PLA+CF_1T301, PLA+CF_1T302, PLA+CF_1T303, PLA+CF_1T304, and PLA+CF_1T305), designated as Case 24.

The tensile strength also shows notable improvements with increasing infill density. In Case 21, the average tensile strength is 20.67 MPa. This increases to 21.88 MPa in Case 22, reflecting a 5.85% rise. Further improvement is observed in Case 23, where tensile strength reaches 27.43 MPa, a 32.70% increase over Case 21. The highest tensile strength is in Case 24, at 35.40 MPa, marking a 71.32% increase from Case 21. Comparing Case 22 to Case 23, the tensile strength increases by 25.39%, and from Case 22 to Case 24, it rises by 61.80%. The increase from Case 23 to Case 24 is 29.10%.

The strain values exhibit minor fluctuations across the different infill densities. In Case 21, the average strain is 3.69%. This value decreases slightly to 3.39% in Case 22, representing an 8.13% reduction. In Case 23, the strain increases to 3.90%, indicating a 5.70% increase from Case 21. In Case 24, the strain further increases to 4.32%, signifying a 17.07% rise from Case 21. Comparing Case 22 to Case 23, there is a minimal increase of 15.04%, while the increase from Case 22 to Case 24 is 27.44%. The strain from Case 23 to Case 24 increases by 10.77%.

**Figure 14:**
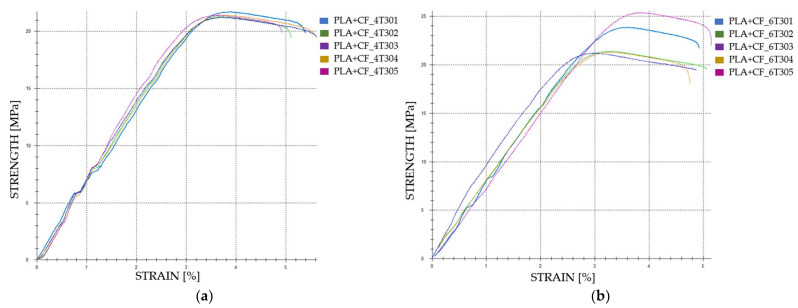

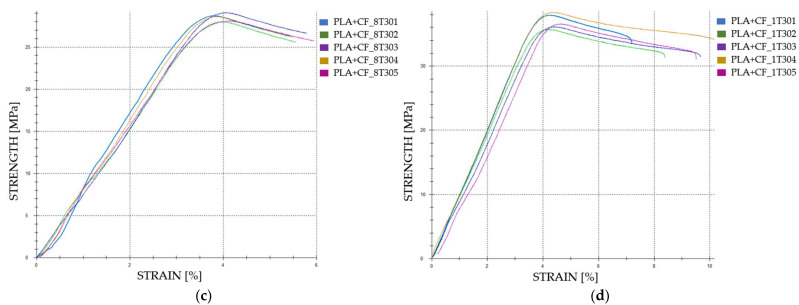
The 3D printed PLA+CF specimens tested: the strength-strain curves: (**a**) PLA+CF tensile-tested specimens with 40% fill density—Case 21; (**b**) PLA+CF tensile-tested specimens with 60% fill density—Case 22, (**c**) PLA+CF tensile-tested specimens with 80% fill density—Case 23, and (**d**) PLA+CF tensile-tested specimens with 100% fill density—Case 24. All the PLA+CF tensile-tested specimens were exposed to cooling lubricant for a period of 30 days.

The graphical representations of the tensile strength and strain for individual FDM 3D-printed PLA+CF tensile-tested specimens that were exposed to cooling lubricant for a period of 30 days are shown in Figure 14.

**Figure 15:**
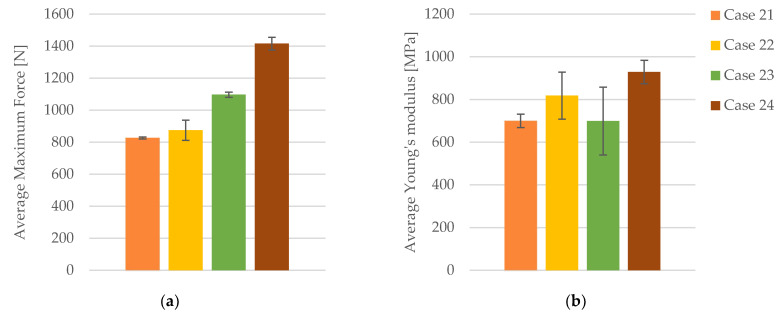
The mechanical parameters of FDM 3D-printed PLA+CF specimens tested that were exposed to cooling lubricant for a period of 30 days: (**a**) average maximum force, (**b**) average Young’s modulus.

The maximum force exhibited by the specimens demonstrates a significant increase with higher infill densities. In Case 21, with a 40% infill density, the average maximum force is 826.93 N (Figure 15a). This value rises to 875.29 N in Case 22, reflecting a 5.84% increase. Further enhancement is observed in Case 23, where the maximum force reaches 1097.05 N, indicating a 32.66% increase compared to Case 21. The highest value is recorded in Case 24, with a maximum force of 1415.94 N, marking a 71.22% increase from Case 21. Comparing Case 22 to Case 23, the maximum force increases by 25.32%, while the increase from Case 22 to Case 24 is 61.75%. The increase from Case 23 to Case 24 is 29.07%.

The stiffness of the material, as indicated by Young’s modulus, exhibits significant increases with higher infill densities. In Case 21, the average Young’s modulus is 700.20 MPa (Figure 15b). This value increases to 818.40 MPa in Case 22, representing a 16.88% increase. In Case 23, Young’s modulus decreases slightly to 699.20 MPa, marking a 0.14% decrease compared to Case 21. The highest value is observed in Case 24, where Young’s modulus reaches 929.00 MPa, signifying a 32.65% increase relative to Case 21. Comparing Case 22 to Case 23, a 14.58% decrease is noted, while the increase from Case 22 to Case 24 is 13.51%. The increase from Case 23 to Case 24 is 32.87%.

The quantitative and qualitative analysis of tensile properties for FDM 3D-printed PLA+CF specimens exposed to cooling lubricant for 30 days clearly demonstrates that increasing the infill density results in significant improvements in maximum force, tensile strength, and Young’s modulus, while exhibiting relatively minor fluctuations in strain.

**Figure 16:**
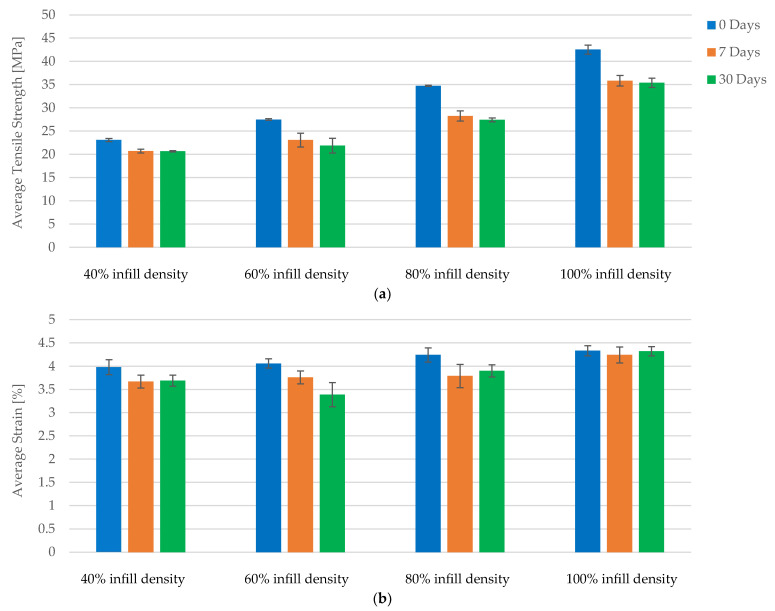
The mechanical parameters of FDM 3D-printed PLA+CF specimens tested: (**a**) average tensile strength, (**b**) average strain.

A comparative analysis of the tensile properties of FDM 3D-printed PLA+CF specimens demonstrates that increased infill density significantly enhances mechanical performance. Across all exposure conditions—no exposure, 7 days of exposure, and 30 days of exposure to cooling lubricant—the data consistently show improvements in maximum force and tensile strength with higher infill densities. Specifically, specimens with 100% infill density exhibit up to a 71.32% increase in tensile strength compared to those with 40% infill density, underscoring the critical role of infill density in enhancing structural integrity and load-bearing capacity (Figure 16a).

The tensile strength data reveal that specimens with 40% infill density have an average tensile strength of approximately 23 MPa without exposure to lubricant. This strength decreases slightly after 7 and 30 days of exposure, stabilizing around 21 MPa. For specimens with 60% infill density, the tensile strength starts at 27.5 MPa and decreases to around 23 MPa after exposure. Specimens with 80% infill density show a decrease from 34.5 MPa to about 27 MPa after exposure, while those with 100% infill density experience a reduction from 42.5 MPa to approximately 35 MPa. These trends highlight that although exposure to cooling lubricant leads to a reduction in tensile strength, higher infill densities still provide superior mechanical performance.

Strain values exhibit minor fluctuations across different infill densities and exposure conditions, indicating stable elasticity. For 40% infill density, the average strain is around 3.9% without exposure, slightly increasing to 4.0% after 7 days and stabilizing at 3.7% after 30 days (Figure 16b). Similar patterns are observed for 60%, 80%, and 100% infill densities, where initial strain values of approximately 4.1%, 4.2%, and 4.3%, respectively, show only slight variations with exposure. The minimal impact on strain suggests that the flexibility of the specimens is retained, even with higher infill densities and exposure to cooling lubricants. This stability in strain is beneficial for applications requiring both strength and flexibility.

The stiffness of the material, as indicated by Young’s modulus, exhibits significant increases with higher infill densities. For 40% infill density, the average Young’s modulus is 652 MPa, decreasing to 504.20 MPa for 60% infill density. However, it increases to 913 MPa for 80% infill density and reaches 1105.80 MPa for 100% infill density. These values indicate a substantial enhancement in rigidity and resistance to deformation with increased infill density. The increase in Young’s modulus from 40% to 100% infill density underscores the importance of infill density in contributing to overall material stiffness, making it suitable for applications where rigidity is essential.

Overall, the quantitative and qualitative analysis of tensile properties for FDM 3D-printed PLA+CF specimens exposed to cooling lubricant for 30 days clearly demonstrates that increasing the infill density results in significant improvements in maximum force, tensile strength, and Young’s modulus, while exhibiting relatively minor fluctuations in strain. Exposure to cooling lubricants does affect mechanical properties, but the benefits of increased infill density are largely retained. This stability under prolonged exposure highlights the suitability of high infill densities for applications in harsh environments where both mechanical strength and resistance to degradation are paramount.

## 4. Discussion

The comparative analysis of the tensile properties of FDM 3D-printed PLA and PLA+CF specimens reveals that increased infill density significantly enhances mechanical performance across all testing conditions. This study systematically examined the effects of varying infill densities and exposure to cooling lubricants over different durations, providing insights into the mechanical robustness and durability of these materials under realistic operating conditions.

**Figure 17:**
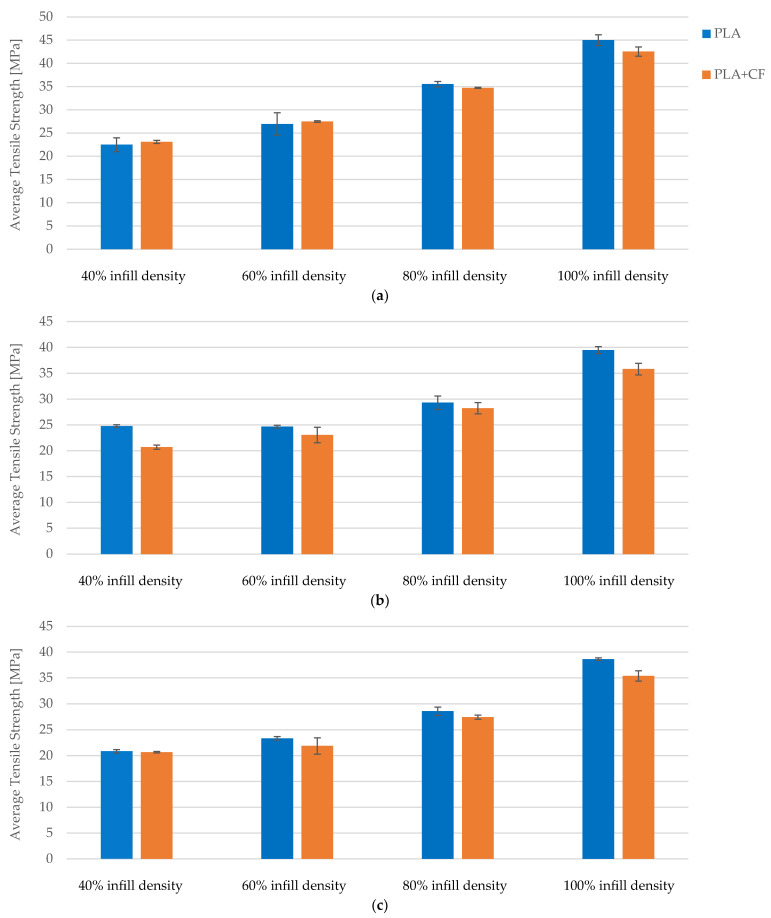
Comparison of tensile strength between PLA and PLA+CF materials at different infill densities and exposure times: (**a**) not exposed to cooling lubricant, (**b**) exposed to cooling lubricant for a period of 7 days, (**c**) exposed to cooling lubricant for a period of 30 days.

The results consistently demonstrate that higher infill densities lead to significant improvements in both tensile strength and maximum force for both PLA and PLA+CF specimens (Figure 17a). For PLA specimens, the tensile strength increased from 22.49 MPa at 40% infill density to 45.00 MPa at 100% infill density, representing a substantial 100.09% enhancement (Figure 17a). Similarly, PLA+CF specimens exhibited an increase in tensile strength from 23.09 MPa at 40% infill density to 42.54 MPa at 100% infill density, marking an 84.27% improvement (Figure 17a). These findings indicate that the structural integrity and load-bearing capacity of the specimens are significantly bolstered by higher infill densities.

The practical implications of these findings are significant for industrial applications of FDM 3D-printed materials. In industries such as medical, and consumer goods manufacturing, selecting the appropriate infill density can improve the durability and performance of 3D-printed components. For instance, in the medical field, prosthetic devices with optimized infill densities can provide better strength and longevity while maintaining the necessary flexibility.

Higher infill densities, although resulting in longer print times and increased material usage, provide substantial benefits in terms of mechanical properties. This trade-off is particularly important in applications where the longevity and reliability of the printed parts are paramount. Additionally, understanding the impact of cooling lubricants on 3D-printed materials helps in making informed decisions about material selection and maintenance procedures in environments where such lubricants are used.

The maximum force showed a corresponding trend, with PLA specimens exhibiting an increase from 899.61 N at 40% infill density to 1800.16 N at 100% infill density. PLA+CF specimens followed a similar pattern, with the maximum force rising from 923.76 N to 1701.51 N. These enhancements underscore the critical role of infill density in improving the mechanical resilience of FDM 3D-printed materials.

Exposure to cooling lubricants over 7 and 30 days slightly diminished the tensile strength and maximum force of both PLA and PLA+CF specimens. Despite this reduction, specimens with higher infill densities maintained superior mechanical properties compared to those with lower infill densities. For instance, after 30 days of exposure, the tensile strength of PLA specimens with 100% infill density was 38.65 MPa, compared to 20.84 MPa for those with 40% infill density. Similarly, PLA+CF specimens with 100% infill density retained a tensile strength of 35.40 MPa after 30 days of exposure, compared to 20.67 MPa for those with 40% infill density (Figure 17c).

**Figure 18:**
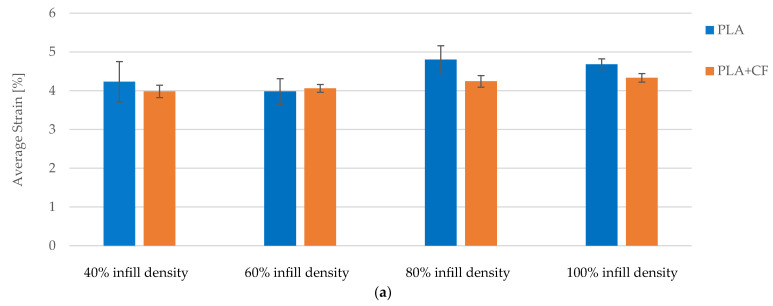

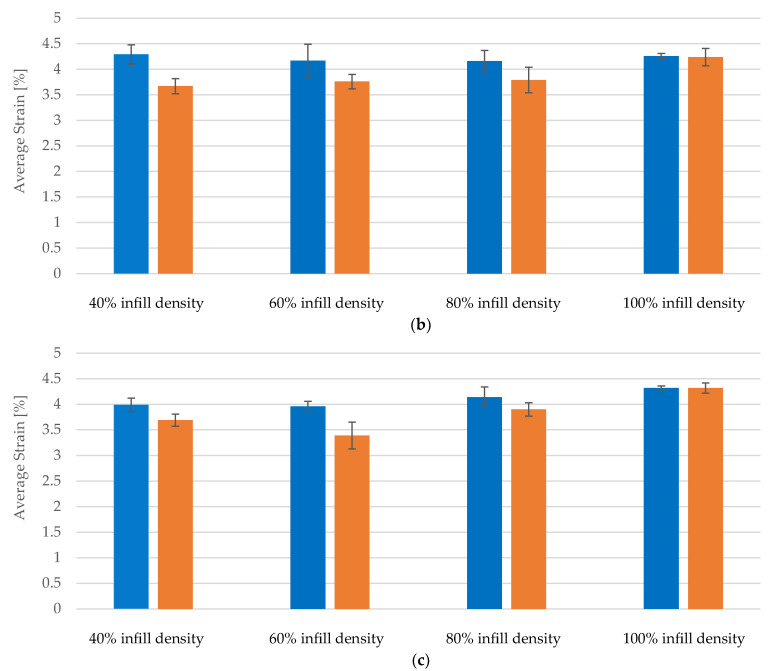
Comparison of strain between PLA and PLA+CF materials at different infill densities and exposure times: (**a**) not exposed to cooling lubricant, (**b**) exposed to cooling lubricant for a period of 7 days, (**c**) exposed to cooling lubricant for a period of 30 days.

Strain values showed minor fluctuations across different infill densities and exposure conditions, indicating stable elasticity (Figure 18). For PLA specimens, strain values ranged from 4.23% at 40% infill density to 4.68% at 100% infill density, showing a slight increase with higher infill densities. PLA+CF specimens demonstrated similar stability, with strain values ranging from 3.98% to 4.33% across the same infill density spectrum (Figure 18a). These results suggest that while higher infill densities enhance mechanical strength, they do not significantly compromise the elasticity of the specimens, which is beneficial for applications requiring both flexibility and strength.

Young’s modulus, a measure of material stiffness, also showed significant improvements with higher infill densities. For PLA specimens, Young’s modulus increased from 595.20 MPa at 40% infill density to 1051.20 MPa at 100% infill density. PLA+CF specimens exhibited a similar trend, with Young’s modulus increasing from 652.00 MPa to 1105.80 MPa. These findings indicate that higher infill densities not only improve strength but also enhance the overall rigidity and resistance to deformation of the materials.

**Figure 19:**
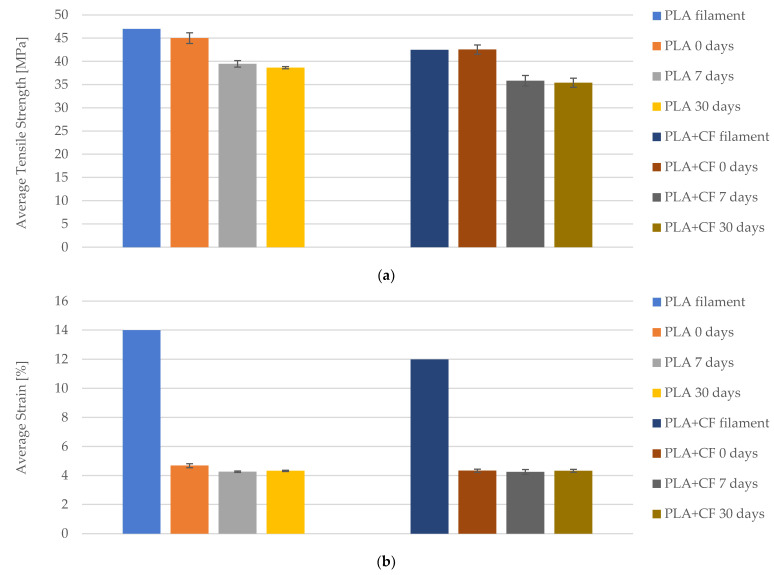
Comparison of tensile strength and strain between PLA and PLA+CF materials with 100% infill densities and exposure times: (**a**) average tensile strength, (**b**) average strain.

The tensile properties of both PLA and PLA+CF materials, under varying exposure times to cooling lubricants, were evaluated through a comprehensive comparative study. Figure 19 presents the graphical representation of average tensile strength and average strain for PLA and PLA+CF materials, each with a 100% infill density, at different exposure intervals: 0 days, 7 days, and 30 days. The data for PLA filament and PLA+CF filament are derived from Table 3, which summarizes the manufacturer’s specifications.

Figure 19a illustrates the changes in average tensile strength for PLA and PLA+CF materials. According to the manufacturer’s data, the PLA filament initially exhibited a tensile strength of approximately 45 MPa. However, after 7 days of exposure to the cooling lubricant, the tensile strength decreased to around 40 MPa, and further declined to 38 MPa after 30 days. This represents a total reduction of about 15.56% over the 30-day period. In comparison, the PLA+CF filament started with a tensile strength of approximately 43 MPa. After 7 days of exposure, its tensile strength dropped to 38 MPa, and after 30 days, it further decreased to 35 MPa, indicating an overall decrease of 18.60%. These trends signify that both materials experience a degradation in tensile strength due to prolonged exposure to the cooling lubricant, with the PLA+CF material showing a slightly higher rate of reduction.

The average strain data, presented in Figure 19b, reveal minor fluctuations across the exposure times for both materials. The PLA filament initially had an average strain of approximately 4.5%, which slightly decreased to 4.0% after 7 days and remained stable at 4.0% after 30 days. Similarly, the PLA+CF filament exhibited an initial strain of around 4.3%, which decreased marginally to 4.2% after 7 days and to 4.0% after 30 days. These findings highlight the relative stability of the elastic properties of both materials despite the observed decrease in tensile strength over time. The consistent strain values suggest that the flexibility of the materials is relatively unaffected by the exposure, which is a crucial factor for applications requiring both strength and elasticity.

The results from this analysis underscore the impact of prolonged exposure to cooling lubricants on the mechanical properties of FDM 3D-printed materials with 100% infill density. Both PLA and PLA+CF materials exhibit a discernible decrease in tensile strength, with PLA showing a 15.56% reduction and PLA+CF an 18.60% reduction after 30 days. This comparative analysis indicates that while carbon fiber reinforcement initially enhances the tensile strength of PLA, it also appears to be more susceptible to the degrading effects of lubricant exposure over extended periods.

The comparative analysis between PLA and PLA+CF specimens highlights the superior mechanical performance of PLA+CF across all conditions. PLA+CF specimens consistently demonstrated higher tensile strength, maximum force, and Young’s modulus compared to PLA specimens. This suggests that the incorporation of carbon fiber significantly enhances the mechanical properties of PLA, making PLA+CF a more robust material for demanding applications.

## 5. Conclusions

This study presents a comprehensive analysis of the tensile properties of FDM 3D-printed PLA and PLA+CF specimens under varying infill densities and exposure to cooling lubricants over different durations. The findings underscore the significant impact of infill density on the mechanical performance of these materials, providing valuable insights into their potential applications in environments that demand high structural integrity and durability.

The results of this study clearly indicate that increasing the infill density of FDM 3D-printed PLA and PLA+CF specimens significantly enhances their mechanical properties, even under prolonged exposure to cooling lubricants. Higher infill densities lead to higher values of tensile strength, maximum force, and Young’s modulus, demonstrating superior material performance. These findings are consistent with previous studies that have also observed improvements in mechanical properties with increased infill density, thereby reinforcing the importance of this parameter in the performance of 3D-printed materials. The strain values exhibited slight increases, suggesting improved elasticity. The stability of these enhanced properties under potentially degrading conditions highlights the suitability of high infill densities for applications in harsh environments where both mechanical strength and resistance to degradation are critical. Future research should focus on long-term environmental effects on mechanical properties and explore further optimization of printing parameters to enhance material performance. This study provides a strong foundation for further research and practical applications of FDM 3D-printed materials in various industries.

## Figures and Tables

**Table 1:** PLA and PLA+CF filaments specifications [55].

Material Type	PLA	PLA+CF
Diameter (mm)	1.75	1.75
Net filament weight (g)	1000	1000
Water absorption (equilibrium in water, 23 °C)	<0.3	0.5
Printing speed (mm/s)	40–60	60–90
Layer height (mm)	0.1–0.2	0.1–0.2
Extrusion temperature (°C)	190–220	200–230
Bed platform temperature (°C)	50–55	40–50

**Table 3:** Main printing parameters, according to [57].

3D Printing Parameter	PLA	PLA+CF
Filament diameter (mm)	1.75	1.75
Infill pattern	Honeycomb	Honeycomb
Infill density (%)	40, 60, 80, 100	40, 60, 80, 100
Nozzle diameter (mm)	0.4	0.4
Base print speed (mm/s)	60	60
Travel speed (mm/s)	100	100
First layer maximum (mm/s)	10	10
Top solid layers	4	4
Bottom solid layers	3	3
Layer height (mm)	0.2	0.2
First layer height (mm)	0.3	0.3
Extrusion temperature (°C)	210	225
Bed temperature (°C)	50	50

**Table 4:** Tensile test results for PLA specimens—not exposed to cooling lubricant.

Case	Specimen Code	Max. Force [N]	Tensile Strength [MPa]	Strain [%]	Young’s Modulus [MPa]
Case 1	PLA_4T1	966.79	24.17	5.00	493
	PLA_4T2	977.09	24.41	4.73	662
	PLA_4T3	855.98	21.40	3.88	595
	PLA_4T4	840.13	21.00	3.72	541
	PLA_4T5	858.06	21.45	3.84	685
	*Average*	*899.61*	*22.49*	*4.23*	*595.20*
	*St. Dev.*	*59.47*	*1.48*	*0.52*	*71.98*
	PLA_6T1	943.49	23.59	3.46	779
	PLA_6T2	976.50	24.41	3.77	871
Case 2	PLA_6T3	1140.88	28.52	4.06	733
	PLA_6T4	1166.76	29.17	4.21	709
	PLA_6T5	1162.77	29.07	4.38	653
	*Average*	*1078.08*	*26.95*	*3.98*	*749.00*
	*St. Dev.*	*97.38*	*2.43*	*0.33*	*73.29*
	PLA_8T1	1444.58	36.11	4.45	771
	PLA_8T2	1438.17	35.95	5.28	529
Case 3	PLA_8T3	1387.41	34.69	4.64	654
	PLA_8T4	1434.09	35.85	5.19	521
	PLA_8T5	1404.10	35.10	4.46	408
	*Average*	*1421.67*	*35.54*	*4.80*	*576.60*
	*St. Dev.*	*22.06*	*0.55*	*0.36*	*124.55*
Case 4	PLA_1T1	1828.56	45.71	4.67	1115
	PLA_1T2	1709.83	42.75	4.42	1095
	PLA_1T3	1834.72	45.87	4.81	977
	PLA_1T4	1809.31	45.23	4.81	950
	PLA_1T5	1818.39	45.46	4.70	1119
	*Average*	*1800.16*	*45.00*	*4.68*	*1051.20*
	*St. Dev.*	*45.99*	*1.15*	*0.14*	*72.57*

**Table 5:** Tensile test results for PLA specimens—exposed to cooling lubricant for a period of 7 days.

Case	Specimen code	Max. Force [N]	Tensile Strength [MPa]	Strain [%]	Young’s Modulus [MPa]
Case 5	PLA_4T71	872.57	21.81	4.47	557
	PLA_4T72	886.77	22.17	4.37	625
	PLA_4T73	875.14	21.88	4.26	609
	PLA_4T74	855.21	21.38	3.95	655
	PLA_4T75	865.55	21.64	4.42	600
	*Average*	*871.05*	*21.78*	*4.29*	*609.20*
	*St. Dev.*	*10.46*	*0.26*	*0.19*	*32.11*
	PLA_6T71	980.39	24.51	4.36	629
	PLA_6T72	901.35	25.03	4.70	473
Case 6	PLA_6T73	990.86	24.77	4.03	695
	PLA_6T74	970.49	24.26	3.82	754
	PLA_6T75	995.09	24.88	3.94	756
	*Average*	*967.64*	*24.69*	*4.17*	*661.40*
	*St. Dev.*	*34.23*	*0.27*	*0.32*	*105.07*
	PLA_8T71	1219.89	30.50	4.40	784
	PLA_8T72	1208.87	30.22	4.33	754
Case 7	PLA_8T73	1199.87	30.00	3.94	861
	PLA_8T74	1158.67	28.97	4.24	793
	PLA_8T75	1076.91	26.92	3.88	780
	*Average*	*1172.84*	*29.32*	*4.16*	*794.40*
	*St. Dev.*	*52.24*	*1.31*	*0.21*	*35.74*
Case 8	PLA_1T71	1612.36	40.31	4.35	1030
	PLA_1T72	1538.22	38.46	4.23	1060
	PLA_1T73	1604.78	40.12	4.26	1070
	PLA_1T74	1578.05	39.45	4.22	1081
	PLA_1T75	1560.62	39.02	4.24	1055
	*Average*	*1578.81*	*39.47*	*4.26*	*1059.20*
	*St. Dev.*	*27.49*	*0.69*	*0.05*	*17.10*

**Table 6:** Tensile test results for PLA specimens—not exposed to cooling lubricant for 30 days.

Case	Specimen Code	Max. Force [N]	Tensile Strength [MPa]	Strain [%]	Young’s Modulus [MPa]
Case 9	PLA_4T301	810.72	20.27	4.14	579
	PLA_4T302	849.30	21.23	3.97	684
	PLA_4T303	831.12	20.78	4.02	639
	PLA_4T304	845.33	21.13	3.76	675
	PLA_4T305	832.08	20.80	4.04	608
	*Average*	*833.71*	*20.84*	*3.99*	*637.00*
	*St. Dev.*	*13.53*	*0.34*	*0.13*	*39.65*
	PLA_6T301	925.61	23.14	3.77	727
	PLA_6T302	929.01	23.23	4.04	685
Case 10	PLA_6T303	960.20	24.01	4.00	705
	PLA_6T304	923.99	23.10	3.97	687
	PLA_6T305	930.85	23.27	4.01	688
	*Average*	*933.93*	*23.35*	*3.96*	*698.40*
	*St. Dev.*	*13.36*	*0.34*	*0.10*	*15.99*
	PLA_8T301	1158.34	28.96	3.99	842
	PLA_8T302	1159.91	29.00	4.19	787
Case 11	PLA_8T303	1150.01	28.75	4.40	733
	PLA_8T304	1166.66	29.17	4.28	769
	PLA_8T305	1077.42	26.94	3.86	735
	*Average*	*1142.47*	*28.56*	*4.14*	*773.20*
	*St. Dev.*	*32.95*	*0.82*	*0.20*	*40.04*
Case 12	PLA_1T301	1540.82	38.52	4.27	1018
	PLA_1T302	1538.58	38.46	4.36	1015
	PLA_1T303	1563.66	39.09	4.31	1052
	PLA_1T304	1547.40	38.68	4.29	1046
	PLA_1T305	1539.02	38.48	4.36	1000
	*Average*	*1545.90*	*38.65*	*4.32*	*1026.20*
	*St. Dev.*	*9.43*	*0.24*	*0.04*	*19.98*

**Table 7:** PLA+CF specimens tensile-test results—specimens that were not exposed to cooling lubricant.

Case	Specimen Code	Max. Force [N]	Tensile Strength [MPa]	Strain [%]	Young’s Modulus [MPa]
Case 13	PLA+CF_4T1	897.76	22.44	3.78	493
	PLA+CF_4T2	933.16	23.33	4.09	693
	PLA+CF_4T3	931.15	23.28	4.13	675
	PLA+CF_4T4	931.31	23.28	3.79	715
	PLA+CF_4T5	925.44	23.14	4.11	684
	*Average*	*923.76*	*23.09*	*3.98*	*652.00*
	*St. Dev.*	*13.26*	*0.33*	*0.16*	*80.60*
	PLA+CF_6T1	1088.01	27.20	4.13	279
	PLA+CF_6T2	1108.25	27.71	4.12	447
Case 14	PLA+CF_6T3	1099.72	27.49	3.86	803
	PLA+CF_6T4	1104.18	27.60	4.09	252
	PLA+CF_6T5	1097.97	27.45	4.10	740
	*Average*	*1099.63*	*27.49*	*4.06*	*504.20*
	*St. Dev.*	*6.82*	*0.17*	*0.10*	*229.12*
	PLA+CF_8T1	1387.45	34.69	4.15	947
	PLA+CF_8T2	1396.61	34.92	4.18	920
Case 15	PLA+CF_8T3	1387.98	34.70	4.10	959
	PLA+CF_8T4	1392.13	34.80	4.53	852
	PLA+CF_8T5	1383.80	34.59	4.24	887
	*Average*	*1389.59*	*34.74*	*4.24*	*913.00*
	*St. Dev.*	*4.39*	*0.11*	*0.15*	*39.29*
Case 16	PLA+CF_1T1	1623.84	40.60	4.31	1074
	PLA+CF_1T2	1724.62	43.12	4.49	1075
	PLA+CF_1T3	1713.50	42.84	4.15	1176
	PLA+CF_1T4	1723.88	43.10	4.39	1091
	PLA+CF_1T5	1721.71	43.04	4.33	1113
	*Average*	*1701.51*	*42.54*	*4.33*	*1105.80*
	*St. Dev.*	*39.04*	*0.98*	*0.11*	*37.84*

**Table 8:** PLA+CF specimens tensile test results—specimens that were exposed to cooling lubricant for a period of 7 days.

Case	Specimen Code	Max. Force [N]	Tensile Strength [MPa]	Strain [%]	Young’s Modulus [MPa]
Case 17	PLA+CF_4T71	825.13	20.63	3.60	714
	PLA+CF_4T72	824.42	20.61	3.96	640
	PLA+CF_4T73	840.55	21.01	3.62	690
	PLA+CF_4T74	848.97	21.22	3.65	681
	PLA+CF_4T75	801.33	20.03	3.53	704
	*Average*	*828.08*	*20.70*	*3.67*	*685.80*
	*St. Dev.*	*16.31*	*0.41*	*0.15*	*25.55*
	PLA+CF_6T71	969.74	24.24	3.81	736
	PLA+CF_6T72	964.35	24.11	3.88	744
Case 18	PLA+CF_6T73	940.71	23.52	3.74	770
	PLA+CF_6T74	933.16	23.33	3.87	711
	PLA+CF_6T75	806.35	20.16	3.50	690
	*Average*	*922.86*	*23.07*	*3.76*	*730.20*
	*St. Dev.*	*59.86*	*1.50*	*0.14*	*27.54*
	PLA+CF_8T71	1050.72	26.27	3.37	905
	PLA+CF_8T72	1132.51	28.31	3.84	818
Case 19	PLA+CF_8T73	1174.76	29.37	4.10	792
	PLA+CF_8T74	1166.20	29.15	3.71	912
	PLA+CF_8T75	1126.73	28.17	3.95	812
	*Average*	*1130.18*	*28.25*	*3.79*	*847.80*
	*St. Dev.*	*73.85*	*1.09*	*0.25*	*50.35*
Case 20	PLA+CF_1T71	1433.15	35.83	4.19	992
	PLA+CF_1T72	1403.24	35.08	4.26	974
	PLA+CF_1T73	1520.31	38.01	4.53	969
	PLA+CF_1T74	1407.82	35.20	4.23	979
	PLA+CF_1T75	1401.42	35.04	3.99	1026
	*Average*	*1433.19*	*35.83*	*4.24*	*988.00*
	*St. Dev.*	*45.03*	*1.13*	*0.17*	*20.48*

**Table 9:** PLA+CF specimens tensile test results—specimens were exposed to cooling lubricant for a period of 30 days.

Case	Specimen Code	Max. Force [N]	Tensile Strength [MPa]	Strain [%]	Young s Modulus [MPa]
Case 21	PLA+CF_4T301	837.09	20.93	3.91	737
	PLA+CF_4T302	822.78	20.57	3.62	677
	PLA+CF_4T303	819.94	20.50	3.68	694
	PLA+CF_4T304	826.53	20.66	3.65	658
	PLA+CF_4T305	828.33	20.71	3.58	735
	*Average*	*826.93*	*20.67*	*3.69*	*700.20*
	*St. Dev.*	*5.86*	*0.15*	*0.12*	*31.38*
	PLA+CF_6T301	921.01	23.03	3.52	778
	PLA+CF_6T302	828.11	20.70	3.25	765
Case 22	PLA+CF_6T303	823.74	20.59	3.01	1037
	PLA+CF_6T304	825.22	20.63	3.37	771
	PLA+CF_6T305	978.38	24.46	3.78	741
	*Average*	*875.29*	*21.88*	*3.39*	*818.40*
	*St. Dev.*	*63.42*	*1.59*	*0.26*	*110.01*
	PLA+CF_8T301	1106.40	27.66	3.74	387
	PLA+CF_8T302	1076.76	26.92	3.90	758
Case 23	PLA+CF_8T303	1117.87	27.95	4.04	737
	PLA+CF_8T304	1104.13	27.60	3.76	829
	PLA+CF_8T305	1080.09	27.00	4.06	785
	*Average*	*1097.05*	*27.43*	*3.90*	*699.20*
	*St. Dev.*	*15.94*	*0.40*	*0.13*	*159.10*
Case 24	PLA+CF_1T301	1455.55	36.39	4.22	987
	PLA+CF_1T302	1370.33	34.26	4.19	958
	PLA+CF_1T303	1383.95	34.60	4.34	865
	PLA+CF_1T304	1470.31	36.76	4.38	974
	PLA+CF_1T305	1399.56	34.99	4.46	861
	*Average*	*1415.94*	*35.40*	*4.32*	*929.00*
	*St. Dev.*	*39.74*	*0.99*	*0.10*	*54.68*

## Data Availability

Data sharing available upon request.
